# Real-world study of bevacizumab treatment in patients with ovarian cancer: a Chinese single-institution study of 155 patients

**DOI:** 10.1186/s12905-023-02329-9

**Published:** 2023-04-13

**Authors:** Nan Zhang, Hong Zheng, Yunong Gao, Tong Shu, Hongguo Wang

**Affiliations:** Gynecology Department, Ministry of Education of People’s Republic of China, Peking University Cancer Hospital and Institute, Key Laboratory of Carcinogenesis and Translational Research, Beijing, 100142 China

**Keywords:** Bevacizumab, Ovarian cancer, Real-world study, Maintenance treatment

## Abstract

**Objective:**

The purpose of this study was to retrospectively assess the pattern, compliance, efficacy and safety of bevacizumab in Chinese ovarian cancer patients.

**Methods:**

We reviewed the clinicopathological data of patients with histologically confirmed epithelial ovarian cancer, fallopian tube cancer and primary peritoneal adenocarcinoma, who were diagnosed and treated at the Department of Gynecologic Oncology of Peking University Cancer Hospital between May 2012 and January 2022.

**Results:**

A total of 155 patients were eventually enrolled in this study, with 77 as first-line chemotherapy (FL) and 78 as recurrence therapy (RT) among which 37 patients were platinum sensitive and 41 were platinum resistant. Among the 77 patients in the FL group, 35 received bevacizumab during neoadjuvant chemotherapy (NACT) alone (NT), 23 received bevacizumab during both neoadjuvant and first-line chemotherapy (NT + FL) and 19 received bevacizumab during first-line chemotherapy alone (FLA). Among the 43 patients of NT and NT + FL groups undergoing interval debulking surgery (IDS), 38(88.4%) patients achieved optimally debulking and 24 (55.8%) patients had no residual disease after IDS. The patients in the FL group had a median progression free survival (PFS) of 15(95%CI: 9.951–20.049) months, and the 12-month PFS was 61.7%. In the RT group, the overall response rate (ORR) was 53.8%. According to multivariate analysis, the patients' platinum sensitivity had a significant impact on the PFS in the RT group. 13(8.4%) patients discontinued bevacizumab due to toxicity. Seven patients were in the FL group while 4 patients were in the RT group. The most common adverse event associated with bevacizumab therapy was hypertension.

**Conclusion:**

Bevacizumab is effective and well-tolerated in the real world setting of ovarian cancer treatment. Adding bevacizumab to NACT is feasible and tolerable. Receiving the regimen containing bevacizumab in the last preoperative chemotherapy did not result in increased intraoperative bleeding of IDS. Platinum sensitivity is the most important factor affecting the effectiveness of bevacizumab in recurrent patients.

## Background

Ovarian cancer is a malignant tumor of the female reproductive tract derived from the epithelium, including ovarian cancer, fallopian tube cancer and primary peritoneal adenocarcinoma, which is the most lethal gynecologic malignancy. With an estimated 313959 new cancer cases and 207252 deaths worldwide in 2020, ovarian cancer is the eighth most frequently diagnosed cancer in women [1]. In 2015, the incidence and mortality rates of ovarian cancer in China reached 52.1/100,000 and 22.5/100,000, respectively [2]. Ovarian cancer develops insidiously and approximately 70% of patients are diagnosed with advanced disease. Currently, optimal debulking surgery with platinum-based chemotherapy remains the standard treatment. For patients with comprehensive tumor metastasis that is unsuitable for primary debulking surgery (PDS), platinum-based neoadjuvant chemotherapy (NACT) followed by interval debulking surgery (IDS) has become an important treatment strategy.

Vascular endothelial growth factor (VEGF) is a protein-signaling molecule that plays a crucial role in angiogenesis. VEGF is highly expressed in epithelial ovarian cancer, making it an exciting therapeutic target [3]. In 2014, bevacizumab was the first anti-angiogenesis agent approved by the US Food and Drug Administration (FDA) for platinum-resistant recurrent ovarian cancer. In 2016, it extended the therapeutic indication to platinum-sensitive recurrent ovarian cancer. In 2018, based on the results from the phase III GOG-0218 and ICON-7 trials [4, 5], it approved bevacizumab as a first-line maintenance therapy for patients with newly diagnosed advanced ovarian cancer with surgery and bevacizumab-containing platinum-based chemotherapy. Bevacizumab is also recommended by National Comprehensive Cancer Network (NCCN) guideline for the treatment of newly diagnosed and relapsed ovarian cancer [6]. However, bevacizumab was not approved by the National Medical Products Administration in China for the treatment of ovarian cancer until November 2021. In addition, the reimbursement of drug costs from medical insurance claims has not yet been initiated. It has limited the use of bevacizumab for ovarian cancer patients in China. Since randomized clinical trials have strict inclusion and exclusion criteria, they often fail to reflect actual clinical practice and outcomes. Real-world studies have become increasingly important in providing evidence of treatment effectiveness in actual clinical practice settings [7] and they allow validation of data already confirmed through clinical trials. The aim of this study was to retrospectively evaluate the pattern, compliance, efficacy and safety of bevacizumab in Chinese patients with ovarian cancer.

## Materials and methods

### Study design and population

**Fig. 1:**
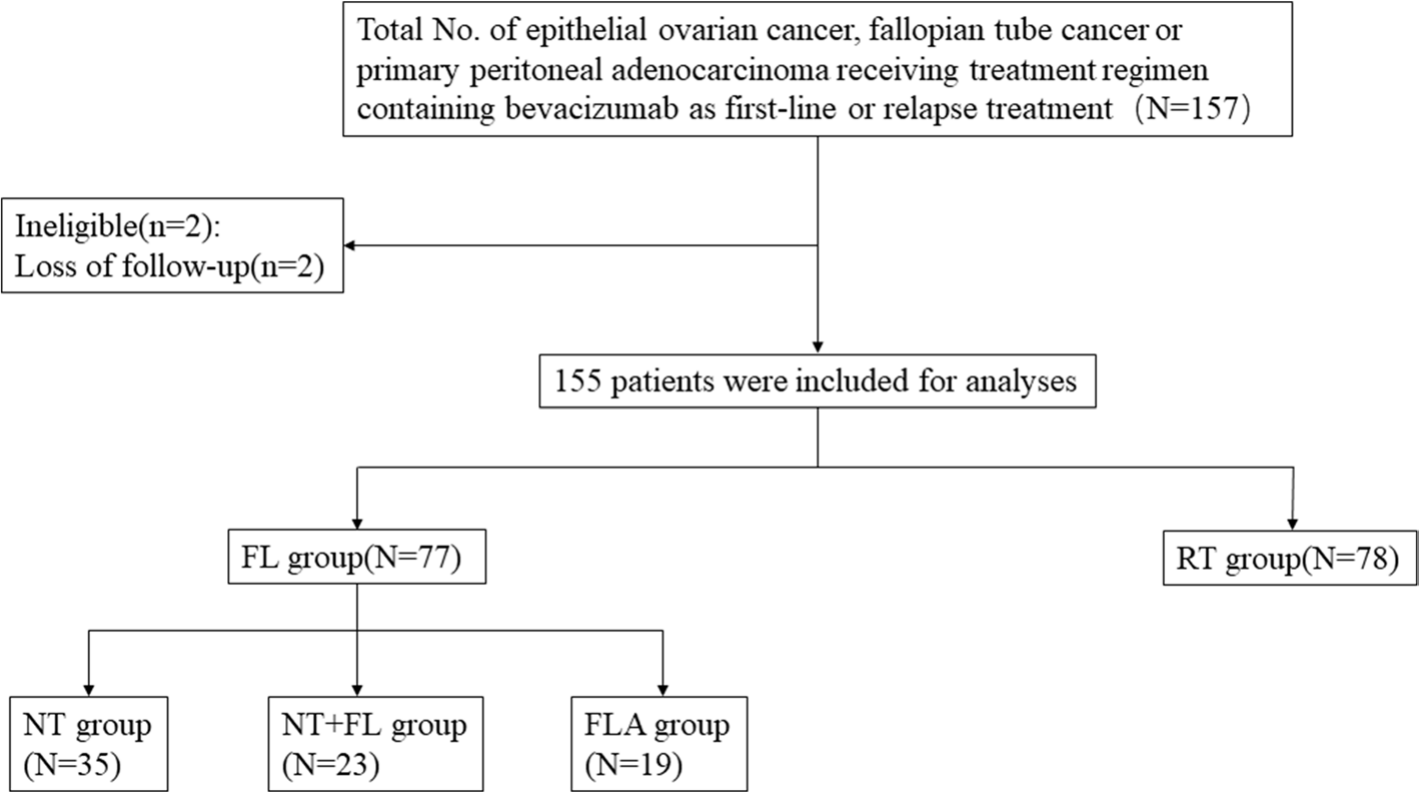
Patients inclusion/exclusion process and treatment pattern of bevacizumab. Abbreviations: FL group, patients receiving bevacizumab as first-line chemotherapy; NT group, patients receiving bevacizumab during NACT alone; NT + FL group, patients receiving bevacizumab during both neoadjuvant and first-line chemotherapy; FLA group, patients receiving bevacizumab during first-line chemotherapy alone; RT group, patients receiving bevacizumab as recurrence therapy

This was a retrospective study and it was approved by the Peking University Cancer Hospital & Institute Review Board (2019YJZ48). We reviewed the clinicopathological data of patients with histologically confirmed epithelial ovarian cancer, fallopian tube cancer and primary peritoneal adenocarcinoma, who were diagnosed and treated at the Department of Gynecologic Oncology of Peking University Cancer Hospital between May 2012 and January 2022. The inclusion criteria of the patients in this study were as follows: 1) at least 18 years old; 2) having pathologically confirmed epithelial ovarian cancer, fallopian tube cancer or primary peritoneal adenocarcinoma; 3) receiving treatment regimen containing bevacizumab as first-line or relapse treatment. Patients were excluded from the study if they lacked follow-up data. A total of 155 patients were included in this study (Fig. 1).

### Outcomes

The efficacy was measured by progression-free survival (PFS) and objective response rate (ORR). Disease response to treatment was assessed according to Response Evaluation Criteria in Solid Tumors (RECIST1.1) [8]. PFS was defined as the duration from the start of treatment until disease progression or death from any cause. ORR was defined as CR or PR. Adverse events (AEs) were recorded according to Common Terminology Criteria for Adverse Events(CTCAE), version 5.0 [9].

### Data collection

Patient data was collected from medical records, including baseline clinical characteristics, treatment patterns, and survival data.

According to the treatment pattern of bevacizumab, patients receiving first-line treatment with bevacizumab were classified into the FL group while patients receiving relapsed treatment with bevacizumab were classified into the RT group. The last follow-up was February 1, 2022.

### Statistical analysis

The categorical variables were described as a percentage, and the continuous variables were described as a median and range. PFS was estimated using the Kaplan–Meier method. A log-rank test was used to evaluate the difference between the two groups. The COX proportional hazards model was used to identify prognostic factors. Prognostic factors with *P* values < 0.1 in univariable analysis were further assessed in multivariable analysis. The SPSS 21.0 statistical package (SPSS Inc., Chicago, IL, USA) was used for statistical analysis.

## Results

### Patient characteristics

A total of 157 potentially eligible patients were screened, and 155 patients were eventually enrolled in this study. Two cases were excluded due to loss of follow-up. Among all involved 155 patients, 77 patients (49.7%, 77/155) were in the FL group; 78 patients (50.3%, 78/155) were in the RT group. Among the 77 patients in the FL group, 35 patients (22.6%, 35/155) were in the NT group, in which patients receiving bevacizumab during NACT alone; 23 patients (14.8%, 23/155) were in the NT + FL group, in which patients receiving bevacizumab during both neoadjuvant and first-line chemotherapy; and 19 patients (12.3%, 19/155) were in the FLA group, in which patients receiving bevacizumab during first-line chemotherapy alone. The details of the clinical characteristics of the involved patients are listed in Table 1.

**Table 1:** Patient characteristics of all 155 included patients

Characteristics	FL group (*N* = 77)			RT group (*N* = 78)	Total (*N* = 155)
	NT group (*N* = 35)	NT + FL group (*N* = 23)	FLA group (*N* = 19)		
Age (years), Median (range)	58(45–71)	59(43–77)	56(36–74)	58(32–73)	58(32–77)
Tumor location, n (%)					
Ovary	35(100)	23(100)	19(100)	77(98.7)	154(99.4)
Fallopian tube	0(0)	0(0)	0(0)	0(0)	0(0)
Primary peritoneum	0(0)	0(0)	0(0)	1(1.3)	1(0.6)
Histology, n(%)					
Serous	35(100)	21(91.4)	18(94.7)	72(92.3)	146(94.2)
Clear cell	0(0)	1(4.3)	0(0)	3(3.8)	4(2.6)
Mucinous	0(0)	0(0)	1(5.3)	2(2.6)	3(1.9)
Others	0(0)	1(4.3)	0(0)	1(1.3)	2(1.3)
Tumor grade, n(%)					
High	34(97.1)	23(100)	18(94.7)	73(93.6)	148(95.5)
Low	1(2.9)	0(0)	1(5.3)	5(6.4)	7(4.5)
FIGO stage, n(%)					
I	0(0)	0(0)	0(0)	2(2.6)	2(1.3)
II	0(0)	0(0)	1(5.3)	7(9.0)	8(5.2)
III	19(54.3)	14(60.9)	12(63.2)	46(59.0)	91(58.7)
IV	13(37.1)	9(39.1)	6(31.5)	22(28.2)	50(32.3)
Unknown	3(8.6)	0(0)	0(0)	1(1.2)	4(2.5)
Administration route, n(%)					
Intravenous	30(85.7)	22(95.7)	19(100)	61(78.2)	132(85.2)
Intraperitoneal	1(2.9)	0(0)	0(0)	8(10.3)	9(5.8)
Intrathoracic	1(2.9)	0(0)	0(0)	2(2.6)	3(1.9)
≥ 2 routes of administration	3(8.5)	1(4.3)	0(0)	7(8.9)	11(7.1)
Dosage (mg/kg), n(%)					
15	7(20)	12(52.2)	8(42.1)	16(20.5)	43(27.7)
7.5	25(71.4)	11(47.8)	11(57.9)	53(67.9)	100(64.5)
5	3(8.6)	0(0)	0(0)	9(11.6)	12(7.8)
Germline BRCA status, n(%)					
BRCA1 mutation	5(14.3)	1(4.3)	1(5.3)	10(12.8)	17(11)
BRCA2 mutation	1(2.8)	1(4.3)	3(15.8)	3(3.8)	8(5.2)
BRCA wild-type	21(60)	20(87.1)	14(73.6)	52(66.7)	107(69)
Unknown	8(22.9)	1(4.3)	1(5.3)	13(16.7)	23(14.8)

*Abbreviations*: *FL group* patients receiving bevacizumab as first-line chemotherapy, *NT group* patients receiving bevacizumab during NACT alone, *NT* + *FL group* patients receiving bevacizumab during both neoadjuvant and first-line chemotherapy, *FLA group* patients receiving bevacizumab during first-line chemotherapy alone, *RT group* patients receiving bevacizumab as recurrence therapy, *FIGO* the International Federation of Gynecology and Obstetrics

All 78 patients in the RT group were treated in combination with other chemotherapeutic or targeted agents. Among them, 37 patients were platinum sensitive and 41 were platinum resistant. 32(41%) patients had received less than three lines of chemotherapy, while 46(59%) had received three or more lines previously.

The follow-up times[median(range)] for the FL group, NT group, NT + FL group, FLA group, RT group were 18(2–84) months, 15(2–48) months, 20(7–49) months, 24(8–84) months and 43(8–158) months, respectively.

The number of courses of bevacizumab[median(range)] for the NT group, NT + FL group, FLA group, RT group were 3(1–20), 8(4–22), 6(1–22), 4(1–12), respectively.

### NACT and first line chemotherapy with bevacizumab

**Fig. 2:**
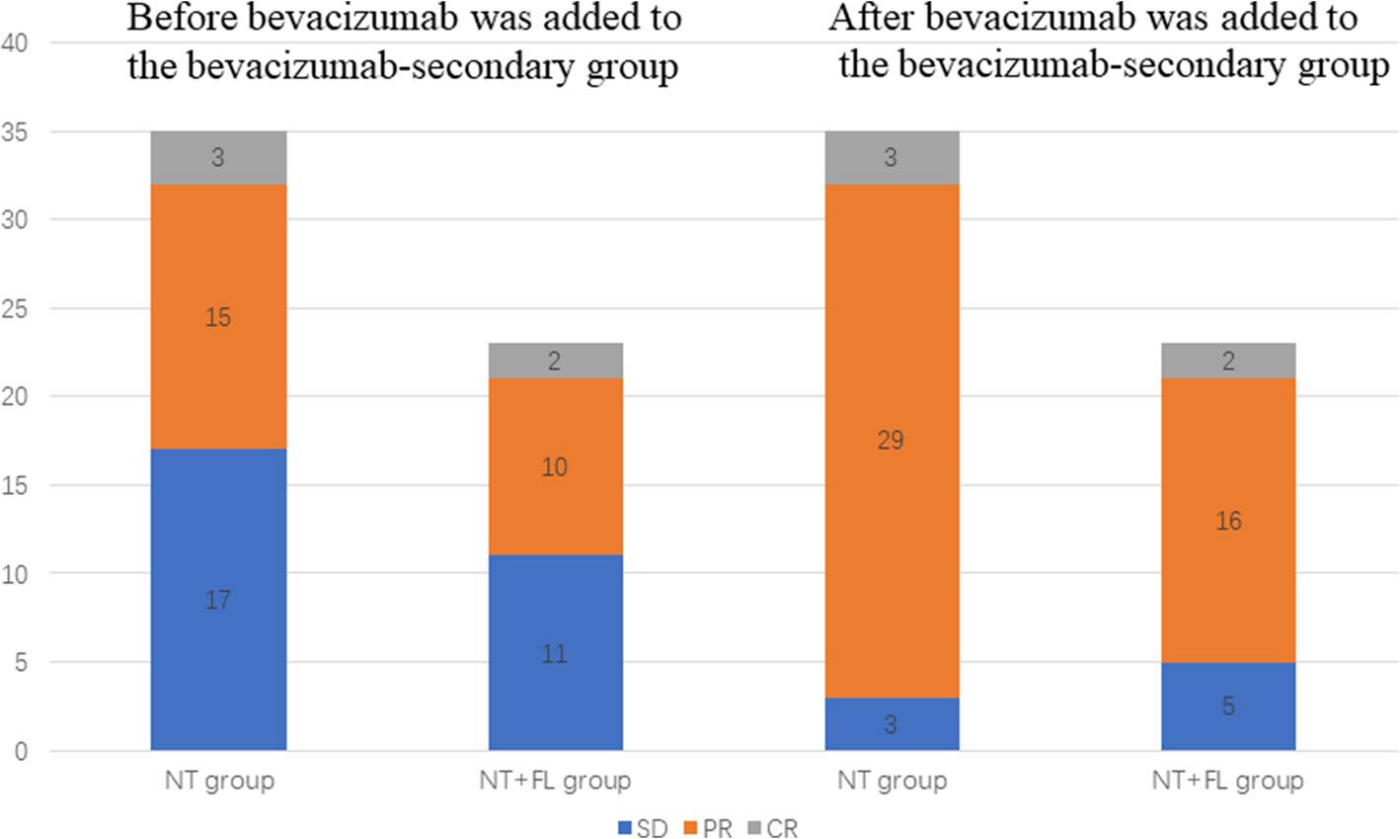
The effect of NACT before and after the addition of bevacizumab to bevacizumab-secondary group

A total of 58 patients in NT group and NT + FL group received NACT containing bevacizumab, among which 33 patients had stage III disease, 23 patients had stage IV disease and 2 patients had unknown status of disease. 69.6% (16/23) of the stage IV patients started chemotherapy with bevacizumab, while only 36.4% (12/33) of stage III patients started chemotherapy with bevacizumab because of extensive tumor spread. Meanwhile, 30.4% (7/23) of stage IV patients and 63.6% (21/33) of stage III patients were added with bevacizumab in their chemotherapy regimen to improve the efficacy after one or two courses of NACT due to a < 50% reduction in CA125 or imaging evaluation of Stable Disease (SD). Among these 28 patients, 10 patients were evaluated as SD by imaging after completing three cycles of chemotherapy, while the CA125 of the remaining 18 patients decreased by less than 50% after completing one cycle of chemotherapy. In the analysis of prognostic factors, these patients were grouped as bevacizumab-secondary, while patients receiving bevacizumab at the beginning of therapy were grouped as bevacizumab-primary. Among the bevacizumab-secondary patients, 17 belonged to the NT group and 11 to the NT + FL group. Of the 18 patients with unsatisfactory CA125 reduction, 14 had a CA125 reduction of more than 50% after a course of chemotherapy with bevacizumab. Ten patients evaluated by imaging as SD were assessed again after two or three courses of bevacizumab with chemotherapy. Six were assessed as PR and four remained SD. Figure 2 shows the effect of NACT before and after the addition of bevacizumab to the bevacizumab-secondary group. For all 58 patients receiving NACT, ORR increased from 51.7% to 86.2% after bevacizumab was added to the bevacizumab-secondary group.

Among the 43 patients undergoing IDS, 38(88.4%) patients achieved optimally debulking that the residual tumor less than 1 cm and 24(55.8%) patients had no residual disease after IDS, including 14 patients (63.6%) in the NT group and 10 patients (47.6%) in the NT + FL group.

Fifteen patients did not receive surgery as a patient was contraindicated due to recent coronary stent implantation; three patients had no visible tumor in the pelvic cavity, for the lesions were predominantly located in lymph nodes of the neck, chest and lung, and reached CR after NACT; seven patients due to personal will; and four patients died of disease progression during NACT.

The median intraoperative blood loss was 200 ml (range 50-1400 ml) of the patients undergoing IDS and 300 ml (range 100-800 ml) in the FLA group. Of the 43 patients undergoing IDS, 28 patients receive the last preoperative chemotherapy without bevacizumab, while 15 patients received the regimen containing bevacizumab. The interval from last bevacizumab to surgery of all the 43 patients was more than six weeks. We didn’t find a significant difference in the intraoperative blood loss between the treatment pattens mentioned above (*p* = 0.717).

In the FLA group, 15 (78.9%) patients had residual disease after primary debulking surgery (PDS).

**Fig. 3:**
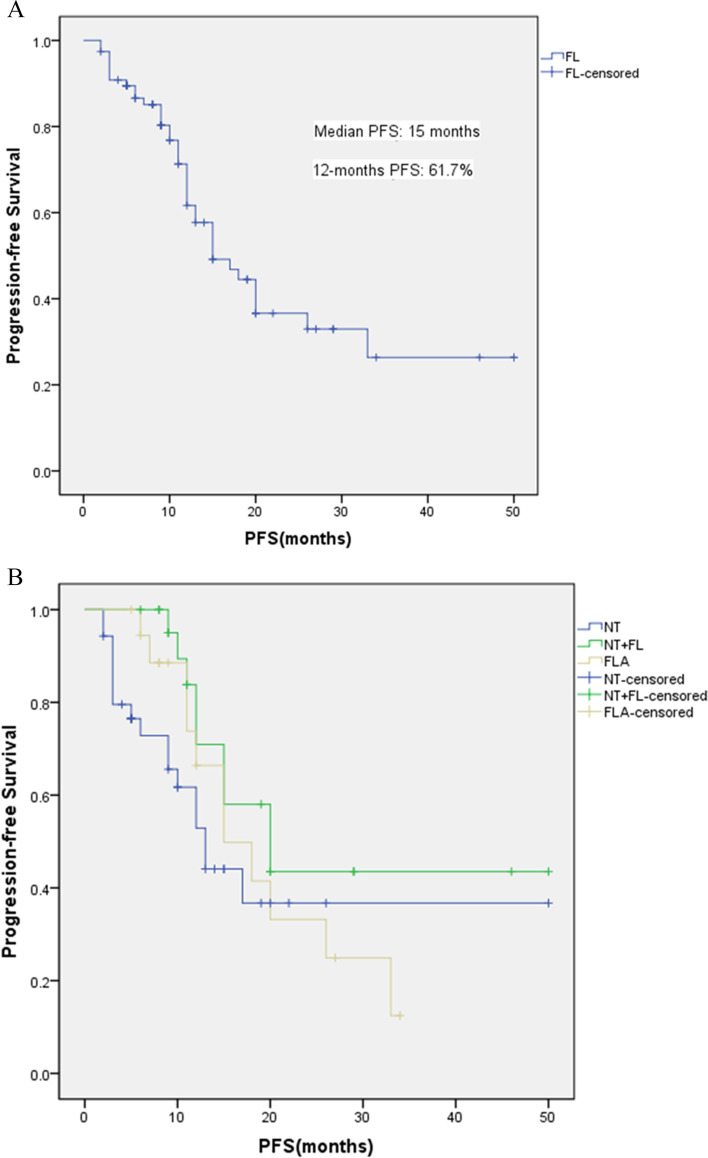
Kaplan–Meier curves for progression-free survival (PFS) in **A**) the FL group, patients receiving first-line treatment with bevacizumab; **B** the three subgroups of the FL group

Figure 3 shows the Kaplan–Meier curves for the PFS of the FL group and the three subgroups. Progression events were observed in 37 patients (48.1%, 37/77), including 17(48.6%, 17/35) in the NT group, 9(39.1%, 9/23) in the NT + FL group, and 11(57.9%, 11/19) in the FLA group. The patients in the FL group had a median PFS of 15 months (95%CI: 9.951–20.049), and the 12-month PFS was 61.7%. The 12-month PFS of the NT group was 52.9%. Nine patients died of disease progression, while one died of cardiogenic disease. In the NT + FL group, the 12-month PFS was 70.9% and three patients died of disease progression. The 12-month PFS of the FLA group was 66.4% and four patients died of disease progression during follow-up.

### Recurrence treatment with bevacizumab

The patients in the RT group had a median PFS of 9 months (95%CI:7.416–10.584), and the 12-month PFS was 23.9%, the ORR was 53.8%, 33 patients died of disease progression during follow-up. The details of the treatment response in the RT group are shown in Table 2.

**Table 2:** The treatment response of 78 patients receiving bevacizumab as recurrence therapy

RT group	No. of patients, n (%)	CR, n (%)	PR, n (%)	ORR, n (%)	SD, n (%)	PD, n (%)
	78(100)	1(1.3)	41(52.6)	42(53.8)	11(14.1)	25(32.1)
Platinum sensitivity						
Sensitive	37(47.4)	1(2.7)	25(67.6)	26(70.3)	3(8.1)	8(21.6)
Resistant	41(52.6)	0(0)	16(39.0)	16(39.0)	8(19.5)	17(41.5)
Number of previous lines of chemotherapy						
< 3	32(41)	0(0)	24(75)	24(75)	4(12.5)	4(12.5)
≥ 3	46(59)	1(2.2)	17(37.0)	18(39.1)	7(15.2)	21(45.7)

*Abbreviations*: *CR* Complete response, *PR* Partial response, *ORR* Objective response rate, *SD* Stable disease, *PD* Progressive disease

#### Prognostic factors

**Fig. 4:**
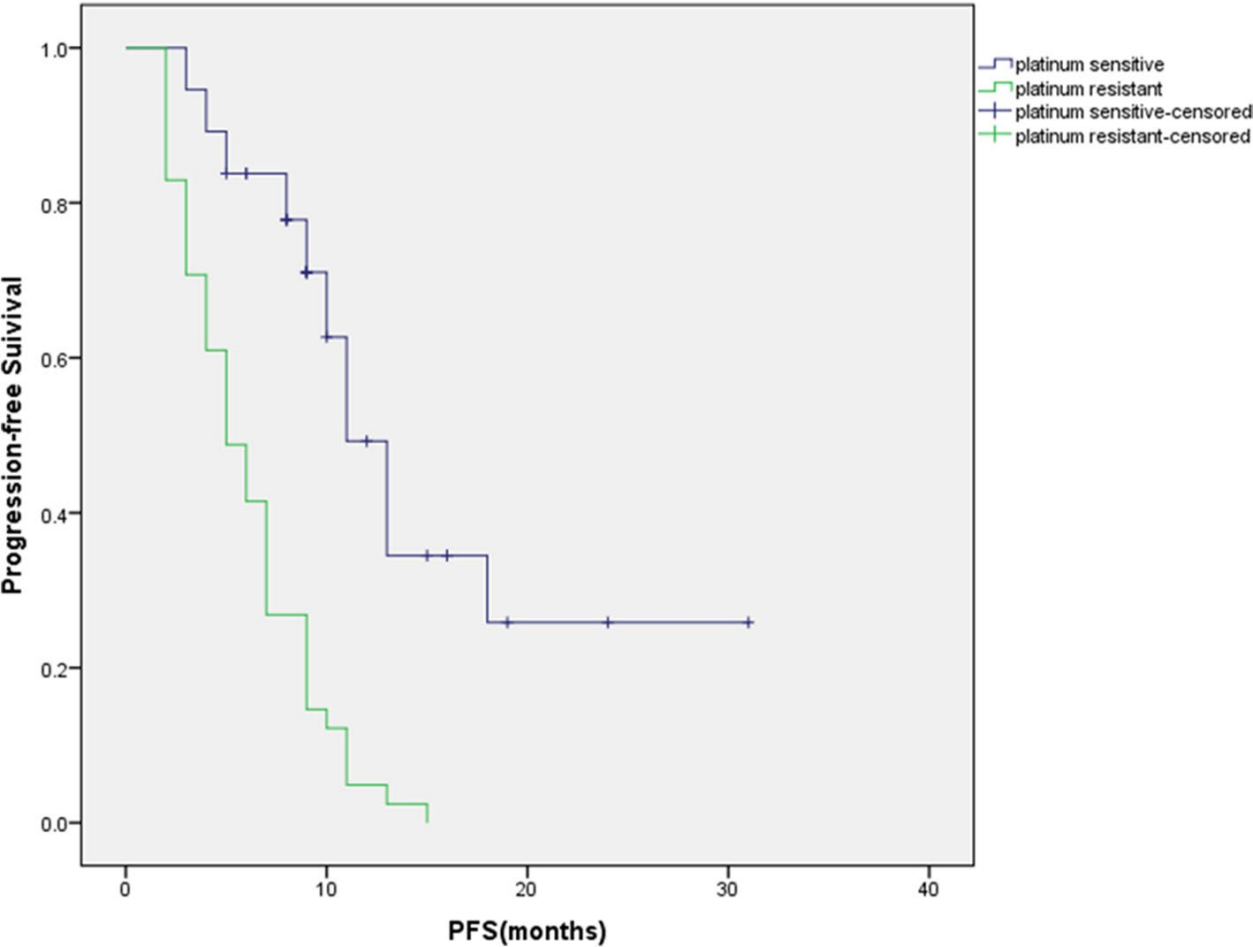
Kaplan–Meier curves for progression-free survival (PFS) of patients in the RT group who were platinum sensitive had a better PFS than those who were platinum resistant

We performed an analysis of age, dosage, complete resection, bevacizumab-primary or secondary and therapy pattern(three subgroups) in the FL group. According to multivariate analysis, no factor was found to affect the PFS in the FL group. In the RT group, we conducted an analysis of age, dosage, platinum sensitivity and previous lines of chemotherapy. The platinum sensitivity of the patients had a significant impact on the PFS in RT group (Fig. 4); The details of the regression results are shown in Table 3.

**Table 3:** Cox regression analysis of factors associated with progression-free survival in the two groups

	Univariate analysis		Multivariate analysis	
	HR(95%CI)	*P* value	HR(95%CI)	*P* value
FL group (*N* = 77)				
Age (< 58 vs. ≥ 58)	0.730(0.381–1.398)	0.342		
Dosage (mg/kg) (7.5 vs. 15)	0.598(0.300–1.193)	0.145		
Complete resection (Yes vs. No)	1.782(0.902–3.520)	0.096	1.949(0.915–4.155)	0.084
Bevacizumab-primary vs. bevacizumab-secondary	1.216(0.625–2.366)	0.564		
Therapy pattern	0.855(0.563–1.298)	0.462		
RT group (*N* = 78)				
Age (< 58 vs. ≥ 58)	0.748(0.450–1.243)	0.262		
Platinum sensitivity (sensitive vs. resistant)	4.080(2.307–7.218)	< 0.001	4.234(2.217–8.084)	< 0.001
Previous lines of chemotherapy (< 3 vs. ≥ 3)	1.426(0.836–2.435)	0.193		
Dosage (mg/kg) (7.5 vs. 15)	0.778(0.419–1.442)	0.425		

#### AEs

Hypertension was the most common AE. 13 patients discontinued bevacizumab due to AEs, including four cases of hypertension, seven cases of thrombogenesis and two cases of impaired wound healing. Among the 43 patients undergoing IDS in the NT group and the NT + FL group, one patient (2.3%) had impaired wound healing. Gastrointestinal perforation or fistula was not observed in this study. No treatment-related deaths or unexpected safety issues were observed. The details of AEs associated with bevacizumab are listed in Table 4.

**Table 4:** Adverse events associated with bevacizumab

Adverse events	Total	G1/2, n (%)	G3/4, n (%)
Total	52(33.5)	41(26.5)	11(7.1)
Hypertension	25(16.1)	16(10.3)	9(5.8)
Proteinuria	9(5.8)	9(5.8)	0(0)
Epistaxis	4(2.6)	4(2.6)	0(0)
Nausea	3(1.9)	3(1.9)	0(0)
Headache	2(1.3)	2(1.3)	0(0)
Thrombogenesis	7(4.5)	7(4.5)	0(0)
Impaired wound healing	2(1.3)	2(1.3)	0(0)

## Discussion

NACT is a therapy strategy for patients with advanced ovarian cancer with the intention of reducing the morbidity of IDS and increasing the probability of optimal debulking. ANTHALYA trial [10] is a multicenter open-label noncomparative Phase II study intended to investigate whether adding bevacizumab to neoadjuvant carboplatin-paclitaxel helps achieve optimal debulking. Ninety-five patients with stage III or IV ovarian cancer were randomized 2:1 to receive four cycles of neoadjuvant carboplatin-paclitaxel with or without bevacizumab during cycles 1–3 before IDS. More patients in the bevacizumab group were candidates for IDS (69 vs. 60%) and the proportion is similar to this study (71.4%). The complete resection rate was 58.6% compared with 46.3% in the Phase III EORTC study [11]. GEICO1205 trial [12] is a randomized Phase II study to evaluate neoadjuvant bevacizumab in newly diagnosed stage III or IV ovarian cancer. More patients receiving bevacizumab than chemotherapy alone underwent IDS (89 vs. 67%, *p* = 0.029). Nevertheless, the complete resection rates were very similar (66 vs.64%, *p* = 0.858). In this study, for stage IV and stage III patients with extensive tumor spread or heavy tumor burden that is difficult to achieve optimal debulking, doctors are more inclined to choose the regimen containing bevacizumab at the beginning of NACT, so as to enhance the efficacy of chemotherapy and surgical resection rate. For patients with SD evaluated after NACT, the addition of bevacizumab to the chemotherapy regimen improved the efficacy and also enabled more patients to have the opportunity for surgery. However, the complete resection rate in this study was somewhat lower than that of the ANTHALYA and GEICO1205 studies, which may be caused by the lower dose of bevacizumab. Of the 43 surgical patients, 26 (60.5%) patients received 7.5 mg/kg dose and 17 (39.5%) received 15 mg/kg dose while the dosage of the above two studies was 15 mg/kg. At the same time, the number of courses of bevacizumab varies widely, as 20 of the 43 surgical patients received only 1–2 courses of bevacizumab in NACT. It also exacerbated the shortage of bevacizumab. Whether increasing the dosage of bevacizumab can improve the complete resection rate needs further study.

To reduce the risk of bleeding and wound healing complications, the drug label for bevacizumab recommends that the drug be stopped at least 28 days before surgery. In this study, for patients with bevacizumab in NACT, doctors commonly performed surgery 6 weeks after withdrawal of bevacizumab without increasing the amount of intraoperative bleeding. We did not find a significant difference in intraoperative blood loss between the treatment patients receiving the regimen containing bevacizumab or not in the last preoperative chemotherapy. As well, prophylactic anticoagulant therapy with low molecular heparin was performed in 39.5% (17/43) of the patients after surgery, and no significant postoperative bleeding was observed. The incidence of impaired wound healing (2.3%) was also similar to that of the new adjuvant chemotherapy cohort in the MITO16A study (3%) [13]. Adding bevacizumab to NACT is feasible and tolerable.

The NT + FL group had a better 12-month PFS than the other two subgroups. We found that the median number of bevacizumab courses in the NT + FL group was eight, and four patients completed 22 treatment courses. While the median numbers of bevacizumab courses were three and six in the NT group and FLA group. Patients in the NT group did not continue to use bevacizumab after IDS, which may be due to: the high proportion of no residual surgery (63.6%) leading to the decrease of doctors' willingness to add bevacizumab in postoperative treatment; six patients who were BRCA mutated received maintenance therapy with a poly-ADP-Ribose polymerase(PARP) inhibitor after chemotherapy, and the choice of another maintenance therapy led to the abandonment of bevacizumab; Bevacizumab was discontinued in one patient due to impaired wound healing and in two patients due to deep vein thrombosis; and economic issues. In the FLA group, 78.9% (15/19) of patients had residual disease after PDS, so doctors would be more likely to use bevacizumab in these high-risk patients. However, in addition to five patients who discontinued bevacizumab due to disease progression, five patients discontinued bevacizumab for economic reasons, and four patients were given PARP inhibitors as maintenance therapy after completing chemotherapy instead of bevacizumab in the FLA group. Inadequate treatment of bevacizumab, premature discontinuation and the use of PARP inhibitors may have an impact on survival.

Currently, with the development of PARP inhibitors in the treatment of ovarian cancer, patients have more options for maintenance therapy. According to NCCN guideline, maintenance therapy with PARP inhibitors may benefit for newly diagnosed stage II-IV high-grade serous carcinoma, G2/3 ovarian endometrioid carcinoma, clear cell carcinoma with BRCA1/2 mutation, and carcinosarcoma after CR or PR is achieved following surgery and platinum-based first-line therapy [6]. In the FL group, 14 patients (18.1%, 14/77) received maintenance therapy with PARP inhibitors instead of bevacizumab after surgery and chemotherapy and BRCA mutations were present in seven of the 14 patients. However, PRAP inhibitors have limited effectiveness for patients with an effective Homologous Recombination Proficient (HRP) mechanism. Whether bevacizumab maintenance therapy is better than PARP inhibitors for HRP patients needs further verification. In addition, bevacizumab requires intravenous administration every 3 weeks and is less convenient than PARP inhibitors, which also reduces patients' willingness to take it. Economic factors were another crucial factor in discontinuing bevacizumab, with 16 patients in the FL group discontinuing it because they could not afford it. It may improve in the future as the price of bevacizumab in China has gradually decreased lately.

The univariate analysis indicated that complete resection had an impact on the PFS (*P* = 0.096) in the FL group. However, according to the multivariate regression analysis, we didn’t find the factor was an independent factor that could impact the PFS (*P* = 0.084). In the FL group, 15 patients did not undergo surgery, which may have diminished the impact of surgery on PFS.

All platinum-sensitive patients in the RT group received platinum-containing chemotherapy combined with bevacizumab. The median PFS was 11.0 months, which is slightly shorter than that of the OCEANS trial (12.4 months) [14] and GOG-0213(13.8 months) [15]. Patients enrolled in the OCEANS trial and GOG-0213 were platinum-sensitive with first recurrence. However, 43.2% (16/37) of the platinum-sensitive patients in this study received three or more prior lines of chemotherapy. We found that the ORR for patients receiving fewer than three lines of previous chemotherapy (75%) was much better than that for patients receiving at least three lines of chemotherapy (39.1%). Besides, the median number of cycles of bevacizumab was six (range:1–12) at time of analysis, fewer than the 12 cycles in the OCEANS trial and the 16 cycles in GOG-0213. These may be the reasons why the median PFS in this study is slightly shorter.

Among the patients with relapse of platinum-resistance, all the other patients were treated with non-platinum single agent combined with bevacizumab except one patient who was treated with Olaparib combined with bevacizumab due to poor chemotherapy tolerance. The median PFS was 5.0 months, which is slightly shorter than that of the AURELIA trial (6.7 months) [16]. The patients in the AURELIA trial received no more than two prior lines of chemotherapy, while 73.2% (30/41) of the platinum-resistant recurrent patients in this study received three or more prior lines of chemotherapy. This may explain the shorter survival.

In addition, the impact of each individual chemotherapy regimen was analyzed in a subsequent analysis of AURELIA trial [17]. The ORR was 53.3% in the weekly paclitaxel with bevacizumab arm and 13.7% in the pegylated liposomal doxorubicin (PLD) with bevacizumab arm. Some phase II trials have been conducted on alternative single-agent chemotherapy combined with bevacizumab regimens. The ORRs were 50% for nabpaclitaxel with addition of bevacizumab [18], 30% for PLD with bevacizumab per three weeks [19], and 33% for PLD with weekly bevacizumab [20]. The ORR of platinum-resistance recurrent patients in this study was 39%. Among these 41 patients, 26.8% (11/41) received nabpaclitaxel as the single-agent chemotherapy combined with bevacizumab, 29.3% (12/41) received PLD regimen, and 14.6% (6/41) received weekly paclitaxel regimen. Which non-platinum single agent combined with bevacizumab has better efficacy in the treatment of platinum-resistant recurrent ovarian cancer needs to be further verified by randomized controlled trials with a larger sample size.

According to our multivariate regression analysis, platinum sensitivity is an independent factor influencing PFS in the RT group. 65.6% (21/32) of the patients received less than three lines of chemotherapy were platinum-sensitive, compared with a platinum sensitivity rate of 34.8% (16/46) for patients with three or more lines of previous chemotherapy. It shows that with the increase in recurrence times, the proportion of platinum-sensitive patients decreased significantly and the proportion of platinum-resistant patients increased, which affected the therapeutic effect.

The safety profile was acceptable in this study as only 8.4% (13/155) patients discontinued bevacizumab due to toxicity. The percentage of ≥ grade 3 AEs was 7.1% (11/155), illustrating that bevacizumab was well tolerated. Most AEs occurred during the combination chemotherapy phase of bevacizumab administration. Similar to some clinical trials [4, 5, 14–16], hypertension was the most common AE and the percentage of hypertension in this study was 16.1%. Other more serious AEs such as gastrointestinal perforation or fistula and central nervous system bleeding were not observed in this study. In the real world, doctors may avoid bevacizumab therapy in patients with bleeding tendencies and gastrointestinal invasion of the tumor. Meanwhile, 64.5% (100/155) of patients received the dosage of 7.5 mg/kg and 27.7% (43/155) received 15 mg/kg. The lower dose may result in a lower incidence of adverse reactions.

As far as we know, no studies of real-world studies of bevacizumab that including the treatment patterns as NACT, first-line, maintenance, and relapse therapy in ovarian cancer patients in China have been published. We researched the prognostic factors and outcomes of 155 patients receiving a treatment regimen containing bevacizumab as NACT, first-line and relapse treatment. However, our study has several limitations, including its single-center, retrospective design. The sample of each treatment group was still small, especially for subgroup analysis. The patients' follow-up time was not long enough to obtain more survival data and analysis. Retrospective follow-up data may result in a lower incidence of AEs than actual.

## Conclusions

Bevacizumab is effective and well-tolerated in the real-world setting of ovarian cancer treatment. Adding bevacizumab to NACT is feasible and tolerable. Receiving the regimen containing bevacizumab in the last preoperative chemotherapy did not result in increased intraoperative bleeding of IDS. Platinum sensitivity is the most important factor affecting the effectiveness of bevacizumab for the recurrent patients.

## Data Availability

The datasets used or analysed during the current study are available from the corresponding author on reasonable request.
